# Current concepts in platelet transfusion

**DOI:** 10.4103/0973-6247.45257

**Published:** 2009-01

**Authors:** Dipika Mohanty

**Affiliations:** *Director (Retd), National Institute of Immunohematology. Mumbai*

**Keywords:** Platelet Rich Plasma, Platelet Transfusion, Hemostatic Factors

## Abstract

This is the era of component therapy. Therefore there is a need for rational use of platelet concentrate. Lot of knowledge has been added recently in the field of platelet specially about the platelet rich plasma and its application in clinical practice. The current review focuses on improvement in preparation of platelet rich plasma, the procedure to make the same more safe and its rational use. Furthermore newer aspects of platelet concentrate use in surgical practice and for regenerative medicine has also been discussed. It also covers some progress and hurdles in preparation of platelet substitutes.

## Introduction

Platelets, although tiny in appearance, are highly active metabolically. Platelets participate in a number of reactions which contribute to maintaining hemostasis in circulation. When activated by exposed sub-endothelium or by circulating agonists, platelets form aggregates which are incorporated into a platelet plug that prevents local hemorrhage. In addition platelets also recruit neutrophils and monocytes by exposing P-selectin on their surface, contribute to signal transmission in neutrophils and endothelial cells by trans-cellular metabolism of released lipid precursors, serve as a site for activated clotting factor assembly and exert a physical force to retract clots. Together, these diverse aspects of platelet physiology make up the clinical efficacy of platelets.

Significant progress has been made in platelet transfusion therapy in the last part of 20^th^ century. In many western countries in the late 1990s, there was decline in the use of red blood cell concentrate, whereas the use of platelet concentrate increased by 80% (Canadian Red Cross Blood Transfusion Service). The disproportionate increase has had considerable effect on the supply of other blood components including the amount of plasma available for fractionation. Of course, India has no data available on this aspect since to date blood banks supplying components are limited. Now two platelet products are available for transfusion, random donor platelets and platelets obtained by apheresis or single donor platelets. This review will mainly focus on the recent developments of the last few years in this field. The best approach will be to focus on specific issues under different headings: (a) Improvement in the preparation of platelet concentrates (b) Platelet derivatives (c) Platelet transfusion (d) Newer use of platelet concentrates.

## Improvement in the Preparation of Platelet Concentrate

- Many modifications in the preparation of platelet transfusions have occurred in recent years. Platelets prepared by standard techniques are contaminated with a significant number of leukocytes. These leukocytes are subsequently responsible for adverse reactions after platelet transfusion. They cause febrile transfusion reactions, adverse immunomodulatory effects, HLA alloimmunization and transfer of some transfusion-associated viruses like cytomegalovirus. Therefore, realizing this problem, transfusion medicine specialists have developed effective means for their removal. These include bedside filtration and pre-storage filtration. Bedside filtration causes about 4 log reduction of WBC on the average. A disadvantage of this method is that it does not remove cytokines or the WBC fragments released during storage. However, pre-storage filtration is able to circumvent these problems. Usually this method removes WBCs from the original whole blood unit within a few hours after collection. Therefore pre-storage filtration is a superior method which produces less febrile reaction due to HLA immunization.[1] Apheresis procedures are available which harvest platelets with reduced numbers of WBC. The WBC content of platelet units as reported by International Society of Blood Transfusion working group should be less than 5 X 10^6^ WBCs in these units usually.
- Additive solution use:
  Attempts have been made to increase the shelf life of the platelet concentrates and maintain their function by addition of synthetic additive solutions.[2,3] The advantages of these non plasma storage media are the prevention of lactate accumulation, removal of products like complement, kallikrin, thrombin and plasmin and removal of platelet micro vesicles.
- Making platelet concentrate safer:
  A major problem with platelets stored at room temperature is the risk of bacterial contamination. Appropriate precautions of course in the collection, preparation and storage reduce the incidence significantly. Leukodepletion also reduces the risk of bacterial contamination. Finally several studies have demonstrated that photo inactivation of platelet concentrate helps in removing some of the viruses. The most promising method is addition of photochemical reagents such as Psoralen compounds that bind to viral DNA. Psoralen is inactivated by UV light of an appropriate wavelength (UVA 320 - 400 nm). The cross-linked product thus produced interferes with viral replication. Novel products are being synthesized with improved DNA binding capacity, requiring shortened period of UVA exposure and causing better platelet preservation.[4] Also some attempt has been made to inactivate HLA antigen by chloroquine treatment for preventing alloimmunization. But this product was found to be toxic. To reduce the incidence of HLA-alloimmunization of transfused patients efforts have been made to inactivate the HLA antigens of platelets by citric acid treatment. However this affects the quality of the platelets.

## Platelet derivatives

### Platelet derived products:

(a) Frozen Platelets (b) Cold liquid platelets (c) Photochemically treated platelets (d) Lyophilized platelets (e) Platelet-derived microparticles (f) Culture derived platelets

### Lyophilized Platelets

Lyophilization was first explored in the 1950s, but animal studies failed to show efficacy *in vivo*. However, in recent years more effective methods have been attempted, which show promise. The platelets were fixed by 1.8% paraformaldehyde, freeze dried with 5% albumin. The rehydrated platelets have similar surface proteins and look like fresh platelets. The adhesive property appears to be maintained. The fixation step kills microorganisms and freeze drying greatly increases the shelf life. They have been shown hemostatically effective in thrombocytopenic animals like rats and rabbits. However, their duration of action seems to be short, and their efficacy in humans has not been tried much.

### Platelet Derived Microparticles

Platelet microparticles are microvesicles of platelet membranes which are formed spontaneously during storage. The microparticles have similar hemostatic property as intact platelets. They can be produced from outdated platelets from blood banks. The product has a shelf life of over 2 years when stored at 4°C. Phase I and II trials have shown to be safe and effective so far.

Platelet derived microparticles are one of the products explored for their clinical use as platelet substitute.[5] Among its greatest advantages is its potential benefit to patients who are refractory and unresponsive to conventional platelet transfusions. This product is prepared by removal of RBCs, WBCs and plasma, and platelets are disrupted by freeze thaw method. After removal of the cellular elements, the product is heated at 60°C for several hours and sonicated. The final product is a liquid preparation containing sucrose and albumin, which is lyophilized and has a shelf life of more than 36 months at 2 to 8°C.

### Culture derived platelets

In 1995, an attempt was made in the laboratory to grow platelets from megakaryocyte progenitors. The resulting platelets appeared to be functionally and morphologically similar to fresh platelets. However, little work has been done to follow up this research.

### Platelet Substitutes

- Red cells with surface bound fibrinogen or RGD peptides.
- Fibrinogen-coated albumin microcapsules / microspheres.
- Liposome-based agents.

One hurdle to the development of new platelet products and platelet substitutes is to define a way to quantify the effects of platelets and related products on bleeding. Several platelet substitutes look promising. Given the demand for platelets and the extremely short shelf life of fresh platelets, there is a real incentive to produce a safe alternative.

Ideally the platelet substitute would have to meet following criteria:

(a) Effective hemostasis with a significant duration of action, (b) No associated thrombogenicity, (c) No immunogenicity, (d) Sterility, (e) Long shelf life with simple storage requirements, (f) Easy preparation and administration.

## Novel Hemostatic factors

Attempts were made in the past to make synthetic platelet analogs to increase and extend the function of autologus platelets in circulation. One of them was thrombospheres, which consist of biodegradable mesospheres of cross-linked albumin coated with fibrinogen, which cross-link to and co-aggregate with platelets.[6,7] In addition, they produce red cells covalently bound to their surfaces which are capable of supporting coagulant activity. Some other investigators also produced red cell coated with peptides containing the Arg-Gly-Asp (RED) which can interact with the activated platelets.[8] Liposome-based platelet substitutes have also been produced which incorporate an extract of platelet membrane into unilamellar lipid vesicles.[9]

Several different forms of platelet substitute are now under development: infusible platelet membranes (IPM), thrombospheres and lyophyilized human platelets. Only the product, IPM is currently in clinical trial in the USA.

### Infusible platelet membranes

They are prepared from outdated human platelets. Then they are fragmented, virally inactivated, lyophilized and can be stored for up to two years. Although the platelet membranes still express some blood group and platelet antigens they appear to be resistant to immune destruction. One IPM product is currently in Phase II trials. The product has successfully stopped bleeding in 60% of thrombocytopenic patients. Overall the product appears to be safe. No adverse effect is noted with no thrombogenecity. Now, they are trying to see the efficacy of this product in cases refractory to platelet transfusion that have developed antibodies to HLA and platelet antigens.

### Thrombospheres

Thrombospheres are not platelets; they are composed of cross-linked human albumins with human fibrinogen bound to the surface. The mechanism of action is not yet elucidated. Experimentally they appear to enhance platelet aggregation but do not themselves activate platelets. They have been shown so far to have no thrombogenicity. One of them has entered clinical trials in Europe.

### Liposome based agents

Two liposome-based agents have been studied with variable success.

(a) Plateletosomes (b) factor Xa with phospholipids vesicles.

Plateletsomes are lipid vesicles with platelet glycoproteins on their surface. But unfortunately although both have been shown to be hemostatically effective in vitro and in some animal models, the second approach has shown high toxicity.

## Platelet Transfusion

### Appropriate use of platelet concentrate

The decision to transfuse platelets depends upon the clinical condition of the patient, the etiology of thrombocytopenia, the platelet count and the functional ability of patients' platelets. The strategies vary from hospital to hospital and also between doctors. It has been reported in the past from Western Ontario that the cardiovascular service used the largest proportion of platelet units (28%), aorta coronary bypass grafting being the most common procedure.[10] Current medical literature supports the appropriate use of platelet concentrate in patients with thrombocytopenia due to chemotherapy but prophylactic use of platelet transfusion for patients undergoing cardiovascular bypass procedures is be questioned. Therefore, continued surveillance of use of these reproduces and re-evaluation of the aims of platelet transfusion is essential. Evidence has accumulated to show that serious bleeding usually occurs only when the platelet count is below 10,000 / μl and blood loss increased only when the platelet count reached 5000 / μl.[11] So, now many hospitals and physicians use platelet counts of 10,000 or 5,000 / μl as the indication for transfusion to uncomplicated patients. However, if the patient is febrile or septic, the old trigger of 20,000 / μl should be used. Active bleeding does not occur with a platelet count above 50,000 / μl unless there is a concomitant platelet function disorder. These patients may require platelet transfusion support at any platelet count.

### Platelet dosage

A platelet concentrate containing approximately 0.7 X 10^11^ platelets should cause a platelet count increase of 5000 to 10,000 / μl in an average sized adult. Most institutions have adopted policies for a standard platelet dose of so many units of pooled platelets. Common local practices have included 4, 6 or 8 to 10 units as their “dose” for adults. However, more hospitals are changing guidelines to more appropriately treat patients of different sizes and weights. They have adopted policies to give one platelet concentrate per 10 kg of body weight. This should increase the platelet count by approximately 40,000 / μl. Increasing use of single donor platelet (SDP) is being seen in oncology practice, which is considered equal to 6 to 8 random donor platelet units. One controversy still persisting is the issue of whether it is more economical and cost effective to give ‘low’ or ‘high’ doses of platelets to patients. More recent data has shown that the best strategy is to give a ‘moderate’ dose of 6 single donor units. In general, especially in oncology practice it is preferable to use leukoreduced platelets in patients with hematological malignancies. The rational is to prevent primary alloimmunization to HLA antigens, since many of these patients ultimately will require platelet transfusions. Patients with severely compromised immunogenic function are at risk of transfusion-associated graft versus host disease. Oncology patients receiving chemotherapy and or irradiation fall into this category. Platelet concentrates should be irradiated before transfusing these patients. It is worth mentioning here that when transfusing platelets no other therapy should be given in another IV line. Such therapies include antibiotics or other biochemical agents that may inhibit platelet function and render the transfusion ineffective.

## Newer use of platelet concentrate

### Platelet gel preparation

Platelet gel mimics the final stage in the clotting cascade. The platelet rich plasma, in presence of thrombin activates platelets, converts fibrinogen to fibrin and promotes further platelet aggregation. Calcium chloride is added to counteract the anticoagulant citrate, thus rapidly forming gelatinous platelet rich glue. This can be used by the surgeon to control unsuturable bleeding sites and the diffuse oozing associated with redo open heart patients. For making this preparation some commercial devices like Sequestra 1000 have also been introduced in USA. It is cost effective, easy to make and since it is autologous eliminates the risk of viral transmission related to donor blood products. This has found a place in surgery, ENT and orthopedic surgery[12] and oral surgery, hepatic surgery, neurosurgery, implant surgery, besides major vascular surgery.[13,14] The list of applications of this product is increasing day by day. It has been successfully used in macular hole surgery.[15]

Since PRP is rich in growth factors like IL-6, IL-3, IL-11, TGF - β and PDGF, it has been utilized as an aid to wound healing. Recently, attempts have been made to utilize PRP combined with stromal stem cells of bone marrow to prepare artificial bone to successfully repair the bone defect in the experimental animal.[16] PRP promotion of chondrogenesis in osteoarthritis has been documented in rabbits.[17] Recent studies suggest that PRP can affect inflammation, post-operative blood loss, infections, osteogenesis, wound and soft tissue healing. Although some authors have reported improved bone formation and tissue healing with PRP, others have had less success.[18–20] These varying results again may be due to PRP preparation, different protocol and techniques. Therefore there is a need for standardized techniques and dosage of PRP. Platelet rich plasma also stimulates stem cells.[21] The use of autologous platelet concentrate activated by autologous thrombin (APCt) is effective and safe in the treatment of chronic diabetic foot ulcers.[22]

### Human Thrombin

Alternative to the use of bovine thrombin, this preparation is available but requires added expense. The exudates from the clot contain regenerated autologous thrombin. The possibility of recombinant human thrombin is more positive now.

## Summary and Conclusion

In recent years blood component therapy is the usual practice. Since platelet rich plasma or concentrate have a shorter shelf life and also produce alloimmunization on repeated use, greater efforts have been made to develop platelet substitutes by several ways, which can be used in clinical practice. Some modified products of platelets are also been tried with variable success. To make platelet concentrate safer attempts have been made to inactivate the pathogens like bacteria, viruses as well as protozoa by using Psoralen and UV light treatment. In addition, for making the platelets less immunogenic, HLA inactivation by treating the platelets with chloroquine has some success. Unfortunately this product lacks in quality of platelets because it appears to be toxic. To circumvent some of the problems and risk of platelet concentrate transfusion, numerous attempts have been made to develop platelet substitutes. Of these products one or two are in Phase I and II trials.

Despite advances in blood component preparation and storage the available platelet products are not free from risks to recipients. These include febrile non-hemolytic transfusion reactions, transmission of viral, bacterial and protozoan infections and alloimmunization resulting in refractoriness to future platelet transfusions. Therefore efforts have been made to develop platelet substitutes as well as some modified products of platelets. There is a great deal of success with some products composed of human albumin microcapsules that have fibrinogen immobilized on their surfaces (synthesis TM). The newer use of platelet concentrate include the repair of bone defects when used with autologous platelets, management of diabetic foot ulcer healing, platelet gel preparation to be used in surgery as glue to manage diffuse oozing in open heart surgery, plastic surgery, eye and ENT and neurosurgery. Current medical literature supports the appropriate use of platelet concentrate. In oncology practice especially, leukoreduced platelet units should be used.
